# Cardiac Manifestations in Behçet’s Syndrome

**DOI:** 10.1007/s11926-025-01190-z

**Published:** 2025-07-17

**Authors:** Federica Bello, Giacomo Bagni, Emire Seyahi, Emanuele Chiara, Iacopo Olivotto, David Saadoun, Giacomo Emmi

**Affiliations:** 1 Department of Experimental and Clinical Medicine, University of Florence, Italy and Internal Interdisciplinary Unit, Behçet Centre, Careggi University Hospital, Florence, Italy; 2 Department of Clinical and Biological Sciences, University of Turin, Turin, Italy; 3 Division of Rheumatology, Department of Internal Medicine and Behçet’s Disease Research Centre, Istanbul University-Cerrahpasa, School of Medicine, Istanbul, Turkey; 4 Department of Medical, Surgical and Health Sciences, University of Trieste, Italy, and Clinical Medicine and Rheumatology Unit, Cattinara University Hospital, Trieste, Italy; 5 Meyer Children’s Hospital IRCCS, Florence, Italy; 6 Department of Internal Medicine and Clinical Immunology, Sorbonne University, Groupe Hospitalier Pitié-Salpêtrière, Assistance Publique-Hôpitaux de Paris (AP-HP), 75013 Paris, France; 7 Centre for Inflammatory Diseases, Monash University Department of Medicine, Monash Medical Centre, Melbourne, Australia

**Keywords:** Behçet, Pericarditis, Myocarditis, Aneurysms, Thrombosis, Vasculitis

## Abstract

**Purpose of the Review:**

Behçet’s Syndrome (BS) is a multisystemic vasculitis that can affect the heart, leading to pericarditis, myocarditis, intracardiac thrombosis, endomyocardial fibrosis, valvular dysfunction, and coronary artery disease. This review summarizes the clinical presentation, diagnostic challenges, and therapeutic strategies for cardiac involvement in BS.

**Recent Findings:**

Advanced imaging techniques have revealed subclinical cardiac involvement in BS. Myocardial dysfunction and fibrosis contribute to heart failure and arrhythmias, while intracardiac thrombi often coexist with pulmonary artery involvement. Coronary artery vasculitis and aneurysms may mimic atherosclerotic disease, complicating diagnosis. Biologic therapies, including TNF-α inhibitors, show promise in refractory cases.

**Summary:**

Early diagnosis and immunosuppressive therapy are crucial. A multidisciplinary approach is essential to managing cardiac complications and optimizing patient outcomes. Future research should refine screening protocols and explore targeted immunotherapies for BS-related cardiovascular disease.

## Introduction

Behçet’s Syndrome (BS) is a complex, multisystemic disease, first described in 1937 by the Turkish dermatologist Hulusi Behçet, who identified the combination of recurrent oral and genital ulcers, and uveitis as part of a single disease. Clinical manifestations, besides this triad, include papulopustular and acneiform cutaneous eruptions, joint inflammation, vascular, central nervous system, and gastrointestinal involvement [1–3]. BS is classified amongst “variable vessels” vasculitis, a definition that stresses its atypical clinical features: veins and arteries of all sizes can be simultaneously affected by an “inflammatory” thrombotic process, with a unique tendency for arterial aneurysm formation [1].

**Fig. 1:**
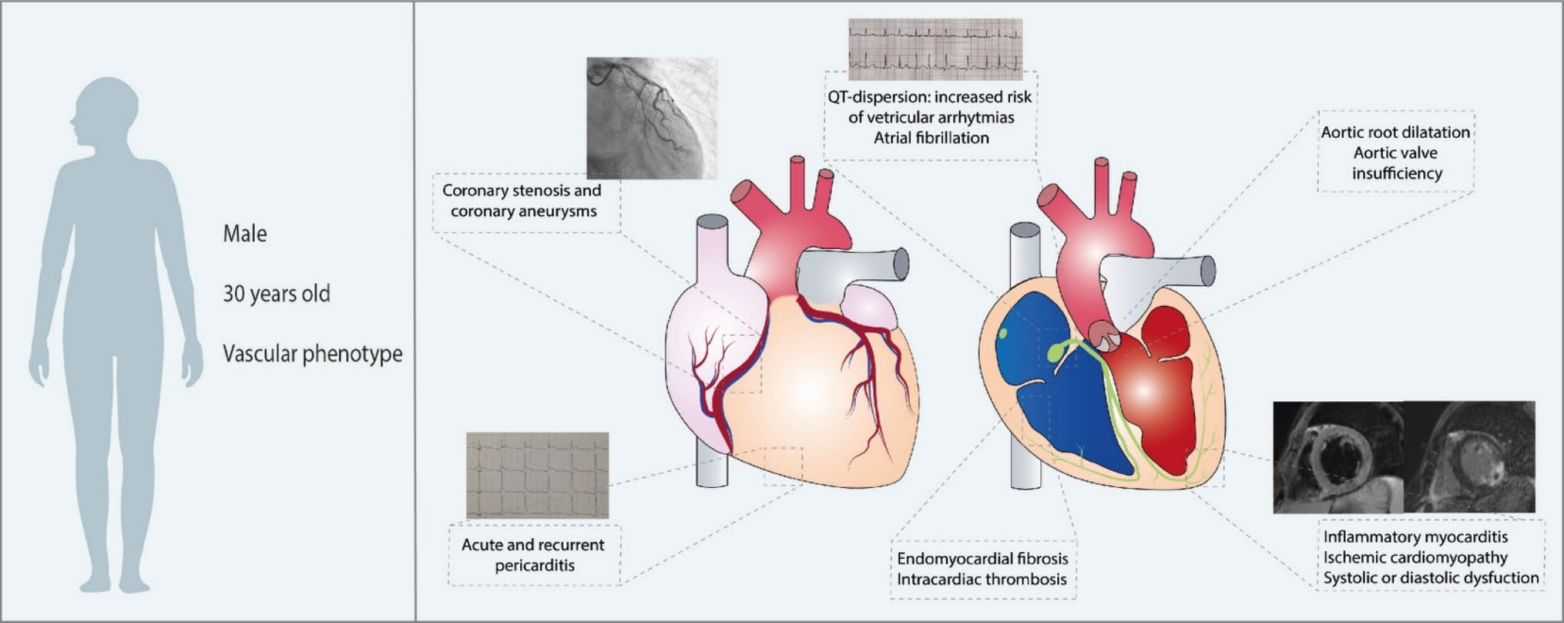
Overview on cardiac involvement in Behçet’s Syndrome. Cardiac manifestations affect mostly young male patients, with a clinical history of vascular manifestations. Cardiac involvement in Behçet Syndrome can present with multiple manifestations, potentially involving all structures of the heart

Cardiac involvement in BS is considered a rare manifestation, with an overall prevalence lower than 5% [4]. However, the actual frequency of heart involvement in BS may be underestimated. Indeed, cardiac lesions were found in 16% of patients in a post-mortem autopsy study in a Japanese BS population [5], and more recently, subclinical cardiac involvement has also been demonstrated by cardiac imaging studies [6]. Males are more often affected than women, at an average age of 30 years, and mortality can reach 20% of affected patients in the months or years following the diagnosis [4]. Cardiac involvement can affect all three tunics (epicardium, myocardium, and pericardium) in the form of endocarditis, myocarditis, pericarditis, intracardiac thrombi, endomyocardial fibrosis, valvular dysfunction, coronary arteritis, or coronary aneurysms [7, 8] (Fig. 1). When present, cardiac involvement can manifest as the starting feature of BS in up to 30% of cases and appears to be strongly associated with vascular thrombosis and arterial aneurysms, constituting the cardiovascular phenotype of BS [9]. Cardiomyopathy in BS can be ischemic, or inflammatory. Clinically, it can manifest as systolic or diastolic heart failure, or as asymptomatic systolic or diastolic dysfunction [10]. Ventricular tachycardia and atrial fibrillation (AF) have been also reported [11], while intracardiac thrombosis represents a classic complication of endocardial involvement.

From a clinical point of view, cardiac involvement in BS can be difficult to diagnose, as signs and symptoms are non-specific and cardiac manifestations are common among the general population. Despite being rare, clinicians should be aware of cardiac involvement in BS, due to its prognostic relevance, also in young patients.

In the present review, we aim at summarizing the current knowledge on cardiac involvement in BS from a clinical, diagnostic, and therapeutic standpoint.

Main studies on cardiac involvement in BS are reported in Table 1, while main cardiac manifestations are summarized in Table 2.

**Table 1:** Main studies on cardiac involvement in Behçet’s Syndrome

Authors and date	Objective of the study	Study design	Population	Results
Kirimli et al., 2000 [78]	To evaluate myocardial involvement in patients with BS by measuring QT-dispersion and heart rate variability	Prospective	N = 28 BS patients	BS had increased QT dispersion, left ventricular diastolic dysfunction, a high incidence of positive late potentials, more complex ventricular arrhythmias
Yagmur et al., 2011 [21]	To evaluate the LV systolic strain by speckle tracking echocardiography and examine its relationship with NT-proBNP	Prospective	N = 32 BS patients and 27 age-matched controls	Regional and mean longitudinal strain was lower in BS patients NT-proBNP levels correlated with mean LV longitudinal strain
Geri et al. 2012 [4]	To report the main characteristics, treatment, and long-term outcomes of BS patients with cardiac manifestations	Retrospective	N 52 patients with cardiac involvement from a cohort of 806 BS patients	Prevalence of cardiac involvement: 6% Pericardial involvement (n = 20; 38.5%); endocardial involvement (n = 14; 26.9%); ICT (n = 10; 19.2%); myocardial infarction (n = 9; 17.3%); endomyocardial fibrosis (n = 4; 7.7%); myocardial aneurysm (n = 1; 1.9%) Patients were more frequently male (p < 0.01), had more arterial (p < 0.01) and venous lesions (p < 0.01) compared to those without cardiac manifestations
Zhu Y L et al. 2012 [34]	To assess the clinical characteristics and outcome of patients with cardiac BS	Retrospective	N = 20 patients with BS and cardiac involvement	Male (n = 17; 85%) Cardiac manifestations: ICT (n = 7, 35%); aortic regurgitation (n = 13, 65%) Surgical treatment in 8 patients (40%); 5 patients (62.5%) underwent re-operation due to recurrence of thrombus or valvular dehiscence and severe paravalvular leakage
Emmungil H et al., 2014 [92]	To present the clinical characteristics and outcome of patients with BS with ICT	Retrospective	N = 22 patients with BS and intracardiac thrombus	Mean age 29.1 years; male to female ratio 20:2 Presenting symptoms: fever (n = 18, 81%), dyspnea (n = 9, 40%) chest pain (n = 9, 40%); hemoptysis (n = 7, 31.8%) Right heart involvement N = 17 patients (77%); Left heart involvement N = 2 patients (9%); both N = 3 patients (13.6%) Treatment: high dose PDN (n = 22, 100%); CYC (n = 18, 81%); AZA (n = 3, 13.6%); warfarin (n = 8, 36.3%)
Aksu et al., 2015 [30]	To compare the frequency of pulmonary, venous, and arterial involvements in BS patients with ICT and general BS population	Systematic review	N = 93 cases of BS with ICT	The right heart was the most common site of ICT. Pulmonary involvement, venous involvement and arterial involvement were more frequent in patients with ICT
Pu et al., 2018 [55]	To determine the echocardiographic manifestations of BS	Retrospective	N = 63 BS patients with cardiac involvement	N = 47 (74.6%) with valvular lesions N = 41 (65.1%) aortic regurgitation N = 17 (30.0%) aortic dilation or aneurysm, aortic pseudoaneurysm and coronary sinus aneurysm
Chen et al., 2019 [59]	To investigate the clinical features and risk factors of coronary involvement in BS	Retrospective	N = 476 BS patients	Coronary involvement = 19 patients (4%), coronary stenosis N = 13, coronary aneurysm N = 9, coronary occlusion N = 3 Pathergy reaction was an independent risk factor for coronary involvement
Lee et al., 2019 [86]	To evaluate the risk of AF in patients with BS	Prospective	N = 6208 BS patients and 31,040 controls	Incidence of AF = 2.3 per 1000 person-years in BS group, and 1.1 per 1000 person-years in control group. The BS group showed a 1.8-fold higher risk of AF compared to the control group
Ahmed et al., 2021 [6]	To delineate the cardiac MRI appearances of cardiac involvement in BS patients	Prospective	30 BS patients without known cardiac disease	Abnormalities on cardiac MRI present in 20 patients (66.67%) Myocardial oedema n = 3 (10%); LGE n = 1 (3.3%); pericardial effusion n = 3 (10.0%); global hypokinesia n = 6 (20.0%); ICT n = 1 (3.3%); pulmonary artery dilatation n = 4 (13.3%); LV end diastolic volume alteration n = 4 (13.3%); RV end diastolic volume alterations n = 7 (23.3%); aortic regurgitation n = 2 (6.7%), tricuspid regurgitation n = 9 (30%); mitral regurgitation n = 9 (30%); left coronary artery dilatation n = 2 (6.7%); arrhythmogenic right ventricular dysplasia n = 1 patient (3.3%)
Fu et al., 2023 [54]	To investigate valvular involvement in patients with cardiac BS. To find out the risk factors of valvular involvement in cardiac BS	Retrospective	N = 121 BS patients with cardiac involvement	N = 77 (63.64%) with valvular involvement Aortic regurgitation (n = 62, 80.52%) Mitral regurgitation (n = 61, 79.22%) Tricuspid regurgitation (n = 41, 53.25%)

*AF* Atrial Fibrillation, *AZA* azathioprine, *BS* Behçet’s Syndrome, *CYC* cyclophosphamide, *ICT* Intracardiac Thrombosis, *LV* Left Ventricle, *LVEF* Left Ventricle Ejection Fraction, *MRI* Magnetic Resonance, *PDN* prednisone, *RV* Right Ventricle

**Table 2:** Comprehensive summary of cardiac manifestations in Behçet’s Syndrome

Cardiac Phenotype	Clinical Features	Diagnostic Tools/Screening Protocols	Treatment Strategies
Pericarditis	Chest pain, dyspnea, asymptomatic pericardial effusion, recurrence is common	EKG (ST-segment elevation, PR depression), TTE (effusion evaluation), CMR	NSAIDs, colchicine, corticosteroids, immunosuppressive agents (azathioprine), biologics (anti-IL1 in refractory cases)
Myocarditis	Systolic or diastolic heart failure, asymptomatic dysfunction, ventricular tachycardia, atrial fibrillation	Cardiac troponin and NT-proBNP levels, TTE, CMR, speckle tracking, PET/CT, histological analysis	Symptomatic therapies (diuretics, beta-blockers, angiotensin-converting–enzyme inhibitors), corticosteroids, DMARDs
Intracardiac Thrombosis (ICT)	Acute presentation with fever, hemoptysis, dyspnea, chest pain, associated with pulmonary artery thrombosis or aneurysms	TTE, transesophageal echocardiography, CTA, CMR, histological analysis	Glucocorticoids, DMARDs (cyclophosphamide, azathioprine), biologics (anti-TNF), anticoagulants, surgical resection if needed
Endomyocardial Fibrosis	Right ventricular involvement, fibrous tissue with calcified areas, potential mural thrombosis	TTE (bright endocardium), CMR (late gadolinium enhancement), angiocardiography	Corticosteroids, DMARDs, surgical excision if heart failure develops
Valvular Involvement	Aortic valve insufficiency, tricuspid valve insufficiency (linked with ICT), mitral valve lesions (rare)	TTE, CMR, surgical exploration if valve insufficiency is severe	Corticosteroids, DMARDs, surgical valve replacement (aortic), careful perioperative immunosuppression
Coronary Artery Disease (CAD)	Acute coronary syndromes, chronic coronary syndromes, coronary aneurysms, low prevalence of classic atherosclerosis	Coronary angiography, CCTA, CMR	Corticosteroids, DMARDs (cyclophosphamide), biologics (anti-TNF), cardiovascular management as per standard protocols
Pulmonary Artery Involvement (PAA/PAT)	Pulmonary artery aneurysms, thrombosis, dyspnea, hemoptysis, high mortality	TTE, CTA, MRI, angiography	Corticosteroids, DMARDs (cyclophosphamide, azathioprine), biologics (anti-TNF), avoid anticoagulation
Arrhythmias	QT dispersion, atrial fibrillation, ventricular arrhythmias, conduction disturbances	EKG, Holter monitoring, electrophysiological studies	Anti-arrhythmic drugs, anticoagulation if AF present, immunosuppressants if inflammatory cause suspected
Aortic Root Disease	Aortitis, aneurysmal changes in Valsalva sinus	TTE, CTA, CMR, aortography, PET/CT	, Immunosuppressive therapy (cyclophosphamide, anti-TNF), Bentall procedure for root replacement

*AF* Atrial Fibrillation, *AZA* Azathioprine, *BS* Behçet’s Syndrome, *CABG* Coronary Artery Bypass Grafting, *CAD* Coronary Artery Disease, *CCTA* Coronary Computed Tomography Angiography, *CMR* Cardiac Magnetic Resonance, *CT* Computer Tomography, *CTA* Computer Tomography Angiography, *DMARDs* Disease Modifying Anti-Rheumatic Drugs, *DVT* Deep Vein Thrombosis, *EKG* Electrocardiography, *ICT* Intracardiac Thrombosis, *IL1* Interleukin-1, *LV* Left Ventricle, *MRI* Magnetic Resonance Imaging, *NSAIDs* Non-Steroidal Anti-Inflammatory Drugs, *NT-proBNP* N-terminal pro b-type Natriuretic Peptide, *PAA* Pulmonary Artery Aneurysm, *PAT* Pulmonary Artery Thrombosis, *PCI* Percutaneous Coronary Intervention, *PET* Positron EmissionTomography, *RV* Right Ventricle, *TNF* Tumor Necrosis Factor, *TTE* Transthoracic Echocardiography

## Pericardial Involvement

Pericarditis has been reported in some series as the most common manifestation of cardiac involvement in BS, accounting for up to 40% of cases [4]. Clinical presentation varies from chest pain and dyspnea to asymptomatic pericardial effusion [12]. Pericardial involvement may be isolated or associated with other types of cardiac involvement, particularly myocardial. The course of pericardial involvement in BS is usually benign, but recurrence is frequent. Serious complications such as cardiac tamponade or constrictive pericarditis are rarely seen [13, 14].

**Fig. 2:**
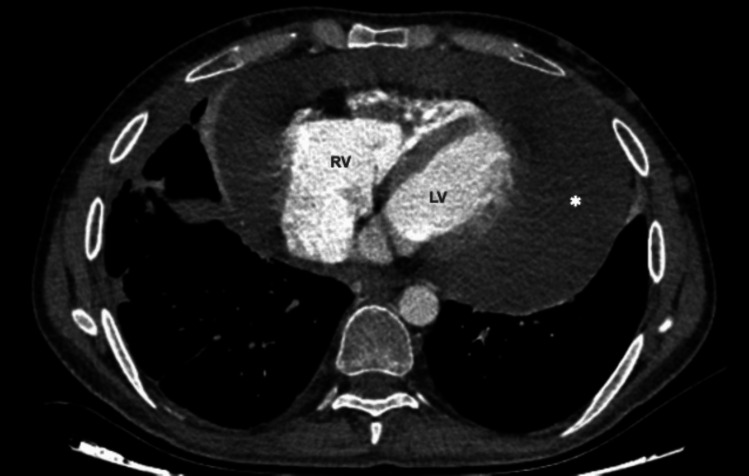
Pericardial effusion in Behçet’s Syndrome: Large pericardial effusion (white asterisk) due to pericarditis and superior vena cava thrombosis in a 31-year-old male. *LV* = *left ventricle; RV* = *right ventricle*

Electrocardiography (EKG) is one of the most useful diagnostic tests for acute pericarditis. It classically shows widespread saddle-shaped or upward concave ST-segment elevation, reflecting subepicardial inflammation, and PR-segment depression, that can accompany or precede ST changes [12]. Laboratory tests should include inflammatory markers (*i.e.,* C reactive protein and erythrocyte sedimentation rate); their elevation is confirmatory, but not diagnostic, as negative blood markers can be seen at the early stages of the disease or after anti-inflammatory therapies. Transthoracic echocardiography (TTE) is commonly used for the initial evaluation and during the follow-up of pericardial disease. TTE provides a semiquantitative estimation of pericardial effusion and assesses its hemodynamic impact. However, TTE has some limitations, including the lack of providing an accurate evaluation of the whole pericardium, from measuring pericardial thickness, to detect loculated effusions. Moreover, TTE cannot assess the nature of pericardial effusion, because of the relative lack of tissue contrast. Computer tomography (CT) and cardiac magnetic resonance (CMR) provide excellent visualization of the pericardium and pericardial space and may have an important role in the assessment of pericarditis and its complications. The definitive diagnosis of pericardial involvement in BS ideally requires documentation of specific histological lesions at pericardial biopsy, such as lymphoplasmacytic or histiocytic vasculitis, fibrinoid necrosis, or granulomatous lesions [15]. However, in clinical practice, a pericardial biopsy is performed extremely seldom, and the diagnosis generally remains presumptive, especially in mild forms, with small-to-moderate effusions and/or with a good response to medical treatment. Red flags suggesting BS pericarditis include large effusions associated with typical signs and symptoms of systemic involvement (Fig. 2).

Nonsteroidal anti-inflammatory drugs (NSAIDs), colchicine, and low-dose corticosteroids constitute the mainstay of medical therapy in BS-associated pericarditis, as for idiopathic—acute or recurrent—forms [16, 17]. Colchicine has been proven effective in preventing relapses and should be considered in cases of pericardial involvement associated with BS [18]. In combination with anti-inflammatory prescriptions, management of the underlying systemic disease is often required, thus immunosuppressive agents, such as azathioprine, may be added for the control of systemic manifestations. Literature regarding the use of biologic Disease Modifying Antirheumatic Drugs (DMARDs) for BS-related pericarditis is scarce: in refractory cases, anti-IL1 agents may be considered [18], while no data exist on anti-Tumor Necrosis Factor (TNF) agents.

## Myocardial Involvement

### Myocardial Dysfunction

Cardiomyopathy can clinically manifest as systolic or diastolic heart failure, or as asymptomatic systolic or diastolic dysfunction [18]. Ventricular tachycardia and atrial fibrillation (AF) have been also reported [19].

In one of the largest studies conducted so far on cardiac involvement in BS by Geri et al., myocardial lesions included myocardial infarcts (17%), and endomyocardial fibrosis (7%), both described more in detail in the following chapters, and left ventricular (LV) myocardial aneurysm (2%) [4].

Inflammatory involvement of the myocardium (*i.e.*, myocarditis) is a rare manifestation of BS [10, 20]; however, the real prevalence may be underestimated as subclinical involvement has been reported. In a cross-sectional observational study of 30 BS patients without known cardiac disease who underwent TTE and CMR, at least one abnormality on cardiac imaging was observed in 20 out of 30 patients (67%) [6]; myocardial edema was observed in 10% and late gadolinium enhancement in 3.3%, while LV and right ventricular (RV) end-diastolic volumes were present in 13.3% and 23.3% patients, respectively. On logistic regression analysis, disease activity, was a significant predictor of cardiac abnormalities [6].

Subclinical systolic dysfunction in BS has also been demonstrated by Yağmur et al., who found an impairment in regional and mean longitudinal strain values, compared to healthy controls, paralleled by an increase in NT-proBNP [21].

Furthermore, in studies that used pulsed wave Doppler tissue echocardiography, ventricular diastolic function was found to be abnormal in BS patients as compared to control subjects [22]. Diastolic dysfunction was reported in up to 37% of patients using TTE and multiplane transoesophageal echocardiography [22]. Similarly, mild systolic and diastolic dysfunction, high frequency of EKG abnormalities, and increased NT-proBNP levels were observed in BS patients with vascular disease with or without pulmonary artery involvement [23]. Potential explanations for systolic and diastolic dysfunction in BS include primary myocardial disease, disturbance of the coronary microcirculation, or the presence of silent ischemia [22].

Corticosteroids and DMARDs are drugs of choice for treating myocarditis [4], along with symptomatic therapies for systolic or diastolic dysfunction. Oral anticoagulants and/or antiplatelets can be used to treat thrombotic complications in BS [4]. However, these therapies should be administered cautiously, given the risk of bleeding, particularly from pulmonary aneurysms.

### Septal Aneurisms

Concerning atrial myocardium, a study on 35 BS patients found no differences in the prevalence of patent *foramen ovale* between BS patients and controls, while a higher incidence of interatrial *septum* aneurysms (31% to 6%) was observed using TTE and multiplane transoesophageal echocardiography [22]. Interatrial *septum* aneurysm has a prevalence of 2–8% in the general population [24, 25] and is associated with an increased risk of cryptogenic stroke and cardiac arrhythmias [26, 27].

## Endocardial Involvement

Endocardial involvement manifests as cardiac valve involvement, intracardiac thrombus, and endomyocardial fibrosis [4]. In the study by *Geri *et al., endocardial lesions are listed as the second most common type of involvement and include intracardiac thrombosis, endomyocardial fibrosis, and cardiac valve lesions [4].

### Intracardiac Thrombosis

**Fig. 3:**
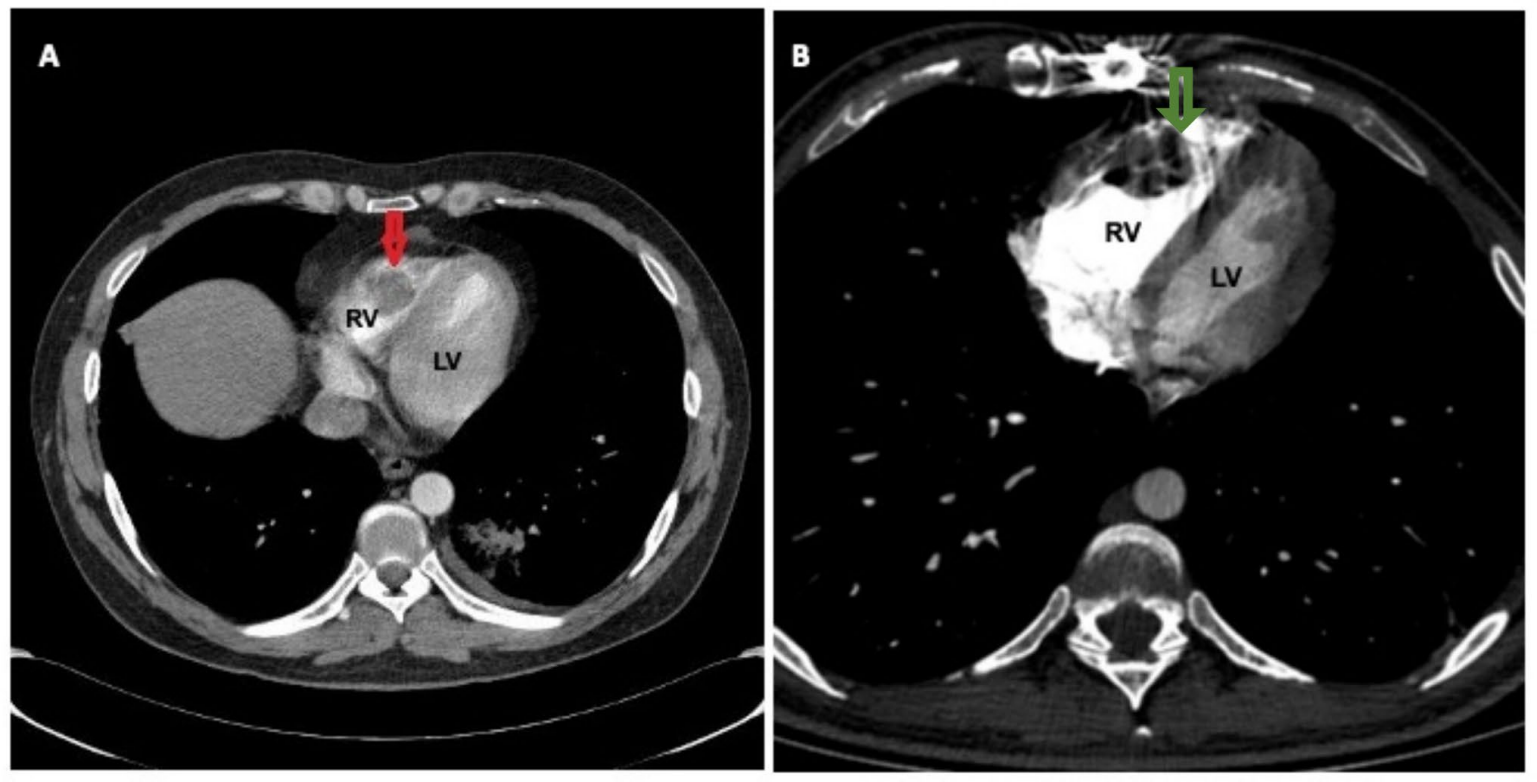
Intracardiac thrombus in Behçet’s Syndrome; **A** Intraventricular filling defect compatible (red arrow) with right ventricle thrombus in a 27-year-old male patient with Behçet’s Syndrome; **B** Thrombus in the right ventricle; the thrombus relapsed after surgical excision (green arrow). See artifacts on the sternum due to the previous sternotomy. *LV* = *left ventricle; RV* = *right ventricle*

Intracardiac thrombosis (ICT) is a serious complication that needs to be managed with a multidisciplinary approach [28] (Fig. 3A and B). The prevalence of ICT is unclear: while a literature review conducted from 1966 to 1999 revealed only 24 cases [29], a more recent literature search from 1996 to 2016 yielded a total of 93 cases [30]. Still, the bulk of the information comes from case reports or series. The majority of the patients are male (87%), usually from Mediterranean basin regions or the Middle East (84%), Turkey being the most common country of origin [29, 30]. The usual onset is in the third decade (mean age: 27 years; range: 12–60 years), although an earlier onset has also been reported [31, 32]. Diagnosis in juvenile cases is particularly challenging, as these patients often might not fulfill the required criteria for BS at presentation [30, 33].

ICT occurs early during the disease course and its presentation is usually acute with fever or increased acute phase response [29]. Fever may be accompanied by hemoptysis, dyspnea, chest pain, and cough most probably due to the concomitant presence of pulmonary arterial involvement [29, 30]. However, in about one-third of the patients, the diagnosis of BS can be delayed because of the absence of typical clinical manifestations (*e.g.,* oral ulcers) [29, 34]. On the other hand, ICT can be difficult to differentiate from infectious endocarditis or myxoma [34]. Angiosarcoma or cardiac lymphoma should be also included in the differential diagnosis. Reports of BS patients misdiagnosed as rheumatic fever despite negative blood cultures or as myxoma are not infrequent [35–39]. Of note, some patients were diagnosed only after exploratory right ventriculotomy and subsequent removal of intracardiac mass [29, 39, 40].

ICT is strongly associated with vascular involvement elsewhere in the body, particularly with pulmonary artery thrombosis (PAT) or aneurysms (PAA). PAT was the most frequent lesion found among 93 BS patients with ICT (52/93, 56%) according to a systematic literature review [30]. The opposite was also true: intra-cardiac filling defects in CT were detected in 28% of 47 patients with PAT or PAA in a single-center follow-up survey [41]. Additionally, ICT can also be associated with deep vein thrombosis (DVT) of the lower extremities, vena cava superior or inferior thrombosis, cerebral venous sinus thrombosis, and Budd-Chiari syndrome [30]. It appears that surgical venous intervention or thrombectomy of a previous DVT, pregnancy, and vaccination against SARS-CoV2 can trigger the formation of intracardiac thrombotic masses [42, 43].

Thrombotic lesions are varied in size, being small or massive, single, or multiple [29, 30, 37]. Importantly, the right side of the heart is more commonly affected than the left side, and the right ventricle more than the right atrium [29]. Right-side predilection could be due to the lower pressure of the right chambers, as well as the right heart being the extension of the vena cava. Lesions are usually immobile and attached to the free wall of cardiac chambers or to the *septum* or to the annulus of the tricuspid valve [29]. Nevertheless, they may extend into the superior or inferior vena cava or protrude through the tricuspid valve. Chronic thrombotic lesions may interfere with the functional integrity of the tricuspid valve [37, 44]. Therefore, thickening, or fibrotic transformation of the tricuspid valve may be observed leading to tricuspid insufficiency and right heart failure despite intensive medical treatment [44]. ICT could be associated with endomyocardial fibrosis, as well [40]. Very rarely, thrombotic mass can attach on the anterior leaflet of the mitral valve [36] and on the pulmonary valve [45].

ICT can be detected using TTE or transoesophageal echocardiography as heterogeneous echogenic masses attached to the endocardium. Lesions can be well visualized by CT or CMR: the latter was found to be more sensitive compared with echocardiography in the visualization of these lesions [46]. Histologically, ICT is characterized by new or organizing thrombus containing dense inflammatory infiltrates, endocardial fibrin deposition, neovascularization, and thick fibrosis [47]. The infiltrate contains primarily neutrophils, followed by lymphoid cells and histiocytes. Less marked inflammatory infiltration can be similarly found within the underlying endocardium and myocardium [47].

The pathologic mechanism of exaggerated thrombotic activity remains largely unknown. Recent developments indicate enhanced neutrophil activity and NETosis implying that the thrombosis in BS is in fact inflammation-induced [2]. Several other mechanisms might have a role in the generation of thrombosis, such as endothelial cell ischemia, disruption or dysfunction, enhanced platelet aggregation, impaired fibrinolysis, increased plasma homocysteine levels, and presence of anti-phospholipid and anti-endothelial antibodies [1, 34]. Systemic glucocorticoids and DMARDs with or without anticoagulation are considered the treatment of first choice. Complete resolution of ICT with immunosuppressive agents has been reported, in the absence of anticoagulation [48–51]. Cyclophosphamide, azathioprine, and anti-TNF agents have been used effectively. Surgical treatment should be reserved for treatment-refractory cases [4] or in those patients with severe tricuspid valve insufficiency [44]. However, recurrences after surgery are common [29]. In the study by *Geri *et al*.*, ICT resolved in 8 out of 10 patients, following treatment with azathioprine or glucocorticoids in addition to anticoagulation [4]. In the remaining 2 patients in whom the thrombus persisted despite treatment, surgical resection was required. Similarly, in two other series, the thrombotic mass disappeared or decreased in size in most of the patients after medical treatment [34, 41]. The mortality rate was reported as 28% (7/25) in a literature survey and the main causes have been attributed to concomitant pulmonary artery involvement and infection [29].

### Endomyocardial Fibrosis

Endomyocardial fibrosis despite being considered a rare complication of BS is reported by several investigators [52] and involves approximately 7% of BS patients with cardiac involvement [4]. Endomyocardial fibrosis is likely the result of vasculitis involving the endocardium, myocardium, or both, and it is often complicated by mural thrombosis [52]. Endomyocardial fibrosis predominantly involves the right ventricle, but may be also located in the left ventricle and—rarely – in the atrium. Echocardiography often displays a bright echogenic endocardium, while CMR shows subendocardial late gadolinium enhancement not restricted to any coronary territory [53]. Both CMR and angiocardiography may show a reduced left or right ventricular size, with enlargement of the respective atrium [53]. Histologically, endomyocardial fibrosis is characterized by dense fibrous tissue with neo-vessels, mononuclear and polymorphonuclear inflammatory infiltrates, and calcified areas.

When endomyocardial fibrosis is complicated by cardiac failure, surgical excision has been reported as successful in the short term [7]. The administration of corticosteroids and DMARDs may deter the development of endomyocardial fibrosis [7].

### Cardiac Valve Lesions

Cardiac valve lesions are present in up to 27% of BS patients with cardiac manifestations [4]. Aortic valve insufficiency is the most common lesion [4, 54] (see under ‘Aortic root involvement’). Tricuspid valve insufficiency occurs as a complication of ICT (see under ‘Endocardial involvement’). Conversely, for unknown reasons, the mitral and pulmonary valves are rarely involved*. Pu *et al*.* studied 97 consecutives patients with BS without already known heart involvement using echocardiography and found that 63 (65%) had at least one cardiac abnormality [54]. Valvular lesions (aortic regurgitation n = 44, mitral regurgitation n = 6) were the most common (75%) cardiac abnormality. Aortic valve lesions were characterized mainly by prolapsed valvular cusps with aortic root aneurysmal changes [55]. On the other hand, mitral valve lesions in BS were characterized by thickened mitral leaflets in the absence of calcification, which is different from what is seen in the classic form of valvular prolapse [55].

## Vascular Involvement Of The Heart

### Coronary Artery Involvement

Coronary artery disease (CAD) in BS requires awareness by the clinician, as it typically affects young subjects and may manifest as ischemic heart disease. Myocardial infarction due to coronary arteritis was reported in up to 17% of BS patients with cardiac involvement [3], and in 25% of cases may be the presenting feature of cardio-BS [56, 57]. It affects mostly adult males, younger than 50 years, with a low prevalence of cardiovascular risk factors [58]. Unlike other systemic inflammatory diseases, such as systemic lupus erythematosus, accelerated atherosclerosis seems not to be a prominent feature of BS [59]. The main mechanism advocated for coronary disease in BS is, as for systemic arteries, vasculitis. Indeed, lymphocytic infiltration of the vessel wall may contribute to fibrotic changes, stenosis, acute thrombotic occlusion, and aneurysm formation. In turn, coronary aneurysms may be asymptomatic [64] or manifest as acute coronary syndromes. Coronary aneurysms are reported in 2% of patients affected with BS [62], but in almost half of the patients with coronary involvement [63]. Aneurysms are often multiple, and most coexist with coronary stenotic lesions [8]. Additionally, extra-cardiac arterial aneurysms and venous thrombotic lesions are frequently reported, suggesting a shared pathogenesis. Histological examination of the affected vascular wall demonstrates an inflammatory obliterative endarteritis of the *vasa vasorum* induced by immune deposition, endothelial cell swelling, and mononuclear perivascular infiltration, with the destruction of the media and fibrosis, resulting in arterial wall weakening and aneurysm or pseudo-aneurysm formation [63].

From a clinical point of view, a retrospective case–control study, identified pathergy reaction as an independent risk factor for coronary involvement in BS [60]. Coronary lesions may manifest through chest pain, dyspnea, epigastric pain, fatigue, syncope, nausea, vomiting, and ventricular arrhythmias; they may be a cause of LV dysfunction, independent of myocarditis. Occasionally, impaired microvascular function [61] or external compression of a coronary artery by aneurismal dilatation of the left Valsalva *sinus* [62], have been described as causes of ischemic manifestations in BS. The prognosis of coronary arteritis and myocardial infarction in BS is poor. In the series by *Geri *et al*.* 7 out of 9 patients with myocardial infarction had a decreased LV function while the remaining 2 died [4].

To date, there is no consensus on the best treatment options for coronary disease management in BS. In general, acute myocardial infarction in patients with BS should be treated according to current guidelines for ischemic heart disease [65]. However, some authors suggest minimizing surgical and invasive procedures and managing CAD medically as much as possible, due to intrinsic tissue frailty derived from vessel wall inflammation [66], which increases the risk of aneurysm and pseudoaneurysm formation [67].

Coronary intervention is also hindered by an increased risk of re-stenosis, as demonstrated in a systematic review exploring outcomes on coronary reperfusion in BS-related CAD. Percutaneous coronary intervention (PCI) was performed in 27% of BS patients with CAD. Of the patients who underwent PCI, stents were placed in 18%, all undergoing re-occlusions. Coronary artery bypass surgery (CABG) was performed more frequently than PCI (35% of cases), with a better outcome, with a single patient needing re-intervention [57].

Besides revascularization strategies and cardiovascular medical treatments, coronary involvement in BS requires immunosuppression for active vasculitis. In a retrospective study, all patients received glucocorticoid treatment and traditional immunosuppressants, with cyclophosphamide, azathioprine, thalidomide, and colchicine being more commonly used [59]. Successful treatment with anti-TNF agents has been reported anecdotally [68], however solid data regarding the efficacy and safety of biologic DMARDs are still lacking.

In young patients, especially males, who present with acute coronary syndrome, it is crucial to investigate additional symptoms that could indicate BS, as a correct diagnosis is essential for receiving the right immunomodulatory treatment and preventing recurrences.

### Aortic Root and Pulmonary Trunk Involvement

Aortic regurgitation is the commonest valvular defect in BS [69]. It is caused by annular dilatation or aneurysm of the Valsalva *sinus*, secondary to aortitis, rather than an intrinsic valvular abnormality [69]. Patients with severe aortic regurgitation may undergo surgical aortic valve replacement; however, the results are often unsatisfactory due to the high rate of treatment failure and post-operative complications secondary to the pathergy phenomenon in the surgical site. In a study *by Ha *et al., valve detachment, suture dehiscence, and perianastomotic leakage were the most common postoperative complications, occurring in almost half of the patients [70].

Lower complication rates are detected in patients who receive intense immunosuppression in the perioperative period. Also, surgical techniques allowing the reinforcement of the vascular wall and the valvular ring implantation to the annulus [71–73] and Bentall-type operations (aortic valve plus aortic root replacement) are associated with more favorable post-surgical outcomes than simple aortic valve replacement [74].

The pulmonary trunk and large pulmonary arteries may be a site of aneurysmal dilatation or, less frequently, PAT (alone or in combination with PAA). Both conditions may be asymptomatic or present with dyspnea or hemoptysis. PAA and PAT are characterized by a mortality greater than 25% [41]. Treatment relies on aggressive immunosuppression mostly with corticosteroids and cyclophosphamide or azathioprine, although anti-TNF agents have been used successfully in refractory cases [41]. After immunosuppression, aneurysms may disappear in a significant proportion of patients, however, the recurrence rate is high, calculated in approximately 20% [50]. Surgery may be considered in patients with giant aneurysms [75, 76], while anticoagulation should be avoided for pulmonary aneurysms and used cautiously in PAT due to the high risk of bleeding.

Overall anticoagulation indications for cardiac manifestations of Behçet’s syndrome are reported in Table 3. It should be emphasized that immunosuppressive therapy remains the cornerstone of treatment for almost every cardiovascular manifestations of BS. Therefore, anticoagulants should be considered only as adjunctive therapy alongside immunosuppressive agents.

**Table 3:** Anticoagulation indications in cardiac manifestations of Behçet’s Syndrome

Context/Condition	Anticoagulation Indication	Preferred Anticoagulant (DOACs vs Warfarin)
Intracardiac Thrombosis (ICT)	Indicated, often combined with immunosuppressive therapy	Warfarin preferred due to lack of evidence on DOACs*
Pulmonary Artery Aneurysm (PAA)	Contraindicated due to risk of bleeding	None
Pulmonary Artery Thrombosis (PAT)	Cautious use, individualized assessment	None if aneurysms are present; Warfarin if thrombosis only
Deep or Superficial Vein Thrombosis (DVT, SVT)	Only in refractory cases (persistence despite adequate immunosuppressive therapy) or concomitant PE	DOACs unless contraindicated; Warfarin
Pulmonary Embolism (PE)	Indicated, combined with immunosuppressive therapy	DOACs unless contraindicated; Warfarin
Atypical Sites Thrombosis	Indicated, combined with immunosuppressive therapy	Warfarin preferred due to lack of evidence on DOACs*
Aortic Root Disease	Indicated if thrombotic involvement is present, combined with immunosuppressive therapy	Warfarin preferred due to lack of evidence on DOACs*
Coronary Artery Disease (CAD)	Indicated only if concomitant atrial fibrillation/flutter (AF)	DOACs unless contraindicated; Warfarin
Arrhythmias	Indicated in AF with high thromboembolic risk	DOACs unless contraindicated; Warfarin
Post-Surgical Valve Replacement	Indicated to prevent valve thrombosis usually combined with immunosuppressive therapy,	Warfarin often preferred; DOACs

*Due to limited data about DOACs use also in the general population in this setting, Warfarin may be preferred

*AF* Atrial Fibrillation; *BS* Behçet’s Syndrome; *CAD* Coronary Artery Disease; *DOACs* Direct Oral Anticoagulants; *DVT* Deep Vein Thrombosis; *ICT* Intracardiac Thrombosis; *INR* International Normalized Ratio, *PAA* Pulmonary Artery Aneurysm, *PAT* Pulmonary Artery Thrombosis, *PE* Pulmonary Embolism, *SVT* Superficial Vein Thrombosis

## Conduction System Involvement and Arrhythmias

Conduction disturbances are uncommonly reported in patients with BS, and usually combined with aortic root dilatation [77] or myocarditis and myocardial fibrosis [78, 79], while left ventricular dysfunction, coronary disease and myocarditis may predispose to ventricular arrhythmias [80]. Several studies on the arrhythmogenic potential of BS evaluated the so-called QT-dispersion (QTd). QTd is defined as the difference between the longest and the shortest QT intervals within a 12-lead EKG. An increase in QTd indicates abnormal ventricular repolarization and has been linked to increased arrhythmogenic potential and sudden cardiac death [81, 82]. The mentioned studies found no statistical difference between the mean QT values in patients with BS and controls, however, ventricular dispersion parameters were significantly longer in cases than in controls [83–86], indicating a potential increase in risk of ventricular arrhythmias and sudden death in BS.

Recently, a nationwide population study by *Lee *et al*.* documented a nearly doubled incidence of AF in patients with BS, compared to the general population [87]. The study provided limited insights into the causative mechanisms underlying AF, however, systemic inflammation is advocated. Indeed, systemic inflammation is a well-known risk factor for AF development in various contexts, and several other inflammatory diseases, such as ankylosing spondylitis and rheumatoid arthritis have been linked to AF occurrence [88, 89]. As a result of the inflammatory processes, electrical and structural remodeling of the atria occurs, providing a substrate for AF development. Patients with BS showed evidence of structural atrial remodeling [89] and increase atrial conduction time [90, 91] compared to controls. Furthermore, in the study by *Lee *et al., patients with more severe disease (*e.g.,* those receiving anti-TNF agents) were more prone to develop AF compared to the non-severe, suggesting again that systemic inflammation might represent a potential risk factor for AF [87].

## Potential Gaps in the Literature

Despite considerable progress in understanding cardiac involvement in BS, several gaps remain in the current literature. The role of biomarkers is increasingly relevant, yet underexplored in the context of BS. NT-proBNP has been mentioned as a marker of myocardial dysfunction, but other cardiac biomarkers, including cardiac troponins, C-reactive protein (CRP), and novel inflammatory markers, have not been thoroughly investigated [93]. Troponins could be valuable in identifying myocarditis or ischemic complications, while CRP might help assess systemic inflammation and stratify cardiac risk [94]. Additionally, novel biomarkers such as cytokines and microRNAs linked to inflammation and thrombosis could offer diagnostic insights, particularly in asymptomatic or subclinical cases.

Another potential gap is the use of PET/CT in assessing cardiac and vascular involvement. While TTE and CMR are standard for detecting structural changes, PET/CT might provide unique insights into inflammatory activity, particularly for coronary or aortic involvement, where conventional imaging is insufficient. Although data are limited, especially for BS, PET/CT could be valuable for detecting vascular inflammation [95].

Long-term cardiac outcomes in BS also remain poorly characterized. Most data are from small, retrospective series with limited follow-up. There is little information on the progression from subclinical to symptomatic cardiac disease, the risk of heart failure, recurrent thrombosis, or sudden cardiac death. Additionally, the long-term efficacy of biologics and the potential need for interventions like cardiac transplantation are not well defined. Prospective studies are crucial to fill these knowledge gaps and establish evidence-based follow-up and management strategies.

Addressing these gaps could lead to more comprehensive diagnostic algorithms, incorporating biomarkers and advanced imaging, as well as more personalized treatment approaches. Improving our understanding of these aspects is essential to optimize the care of patients with cardiac involvement in BS.

## Conclusions

BS is a chronic relapsing systemic vasculitis with multi-organ involvement, and cardiac manifestations can range from subclinical forms to severe conditions. While full-blown cardiac manifestations are known negative prognostic factors, less is understood regarding the prognostic implications of subclinical cardiac involvement and the potential for progression to overt cardiac disease. Early recognition of the disease process is crucial for implementing adequate treatment and preventing complications. Immunosuppressants may improve the prognosis of cardiac manifestations, but the evidence is mostly based on case reports and limited retrospective observations. Also, it remains unclear whether subclinical alterations require early immunosuppressive treatment.

Cardiac involvement in BS is complex and rare, with a lack of consensus on diagnosis and management. The heterogeneity of manifestations makes it difficult to estimate prevalence, potentially leading to an underestimation of cardiac involvement. Prospective multicenter studies are needed to better characterize these manifestations and the long-term outcomes associated with different therapeutic strategies. Standardizing cardiac screening protocols and systematically evaluating the impact of novel therapies, including biologics, could significantly enhance clinical management. Among emerging treatment options, biologic therapies, particularly anti-TNF agents, show promise, especially for refractory or more severe cases, but robust evidence from randomized controlled trials is still scarce. Identifying predictors of therapeutic response and evaluating the efficacy of combination or maintenance regimens will be essential in this context.

In the daily clinical practice, it is also important to consider the role of cardiac screening in patients with BS, implementing routine cardiac evaluation at diagnosis, especially for those with a history of arterial or venous events, and then according to the clinical course of the disease. Maintaining a high index of suspicion for cardiac involvement is crucial, even when symptoms are subtle, and thorough cardiac evaluation is needed in the presence of unexplained dyspnea, chest pain, palpitations, or signs of heart failure.

Lastly, a multidisciplinary approach involving BS-specialists, cardiologists, and imaging specialists is essential for comprehensive assessment and coordinated care. Improving early detection, refining risk stratification, and optimizing therapeutic strategies remain critical priorities for future research, ultimately aiming to enhance outcomes and quality of life of patients with BS.

## Key References

- Geri G, Wechsler B, Thi Huong DL, Isnard R, Piette JC, Amoura Z, Resche-Rigon M, Cacoub P, Saadoun D. *Spectrum of cardiac lesions in Behçet disease: a series of 52 patients and review of the literature.* Medicine (Baltimore). 2012;91[1]:25–34.
  - ⚬ This represents the main and larger study regarding cardiac involvement in Behçet’s syndrome so far.
- Mogulkoc N, Burgess MI, Bishop PW. *Intracardiac thrombus in Behçet's disease: a systematic review.* Chest. 2000;118(2):479–487.
  - ⚬ The study provides a comprehensive overview of the relationship between intracardiac thrombus formation and Behçet’s disease, highlighting the significant cardiovascular risks and guiding clinical management.
- Coşkun S, Ekici Tekin Z, Güngörer V, Çelikel E, Kurt T, Polat MC, Tekgöz PN, Sezer M, Karagöl C, Kaplan MM, Öner N, Gürsu HA, Kavurt AV, Güzelküçük Z, Özbek NY, Çelikel Acar B. *A case series of intracardiac thrombi and vascular involvement in pediatric Behçet's disease.* Rheumatol Int. 2023;43(6):1161-1171.
  - ⚬ This study is relevant because it highlights the occurrence of intracardiac thrombi and vascular involvement in pediatric Behçet’s syndrome, underscoring the need for early diagnosis, tailored management strategies, and heightened clinical awareness in younger patients.
- Zhu YL, Wu QJ, Guo LL, Fang LG, Yan XW, Zhang FC, Zhang X. *The clinical characteristics and outcome of intracardiac thrombus and aortic valvular involvement in Behçet's disease: an analysis of 20 cases*. Clin Exp Rheumatol. 2012;30(3 Suppl 72):S40-S45.
  - ⚬ This study analyzes the clinical characteristics and outcomes of intracardiac thrombus and aortic valvular involvement in Behçet syndrome, providing essential insights for improving diagnosis and treatment strategies in affected patients.
- Huong DL, Wechsler B, Papo T, de Zuttere D, Bletry O, Hernigou A, Delcourt A, Godeau P, Piette JC. *Endomyocardial fibrosis in Behçet's disease.* Ann Rheum Dis. 1997;56(3):205–208.
  - ⚬ This paper explores the association between Behçet’s syndrome and endomyocardial fibrosis, providing insights into its pathophysiology, clinical implications, and potential management strategies.
- Fu J, Liu J, Li X, Tang L, Wu S, Yu H, Zhang C. *Transthoracic echocardiographic assessment of cardiac valves in patients with Behçet's disease*. Int J Cardiovasc Imaging. 2023;39(4):697–706.
  - ⚬ This study is relevant because it demonstrates the utility of transthoracic echocardiography in assessing cardiac valve involvement in Behçet’s syndrome, providing valuable insights into prevalence, diagnostic features, and potential complications.
- Seyahi E, Ugurlu S, Cumali R, Balci H, Ozdemir O, Melikoglu M, Hatemi G, Fresko I, Hamuryudan V, Yurdakul S, Yazici H. *Atherosclerosis in Behçet's Syndrome*. Semin Arhritis Rheum. 2008;38(1):1–12.
  - ⚬ This paper is relevant since it examines the link between Behçet’s syndrome and atherosclerosis, shedding light on vascular involvement, risk factors, and potential mechanisms underlying cardiovascular complications in these patients.
- Kirimli O, Aslan O, Göldeli O, Güneri S, Badak O, Fetil E, Ozkan S. *Heart rate variability, late potentials and QT dispersion as markers of myocardial involvement in patients with Behçet's disease*. Can J Cardiol. 2000;16(3):345–351.
  - ⚬ This article is relevant since it investigates heart rate variability, late potentials, and QT dispersion as markers of myocardial involvement in Behçet’s syndrome, providing valuable insights into its cardiac manifestations and potential arrhythmic risks.
- Lee E, Choi EK, Jung JH, Han KD, Lee SR, Cha MJ, Lim WH, Oh S. Increased risk of atrial fibrillation in patients with Behçet's disease: *A nationwide population-based study*. Int J Cardiol. 2019;292:106–111.
  - ⚬ This article provides population-based evidence of an increased risk of atrial fibrillation in patients with Behçet’s syndrome, contributing to the understanding of its cardiac complications and potential clinical implications.

## Data Availability

No datasets were generated or analysed during the current study.
